# Postpartum Vascular Dysfunction in the Reduced Uteroplacental Perfusion Model of Preeclampsia

**DOI:** 10.1371/journal.pone.0162487

**Published:** 2016-09-22

**Authors:** Lesley Brennan, Jude S. Morton, Anita Quon, Sandra T. Davidge

**Affiliations:** 1 Department of Obstetrics and Gynaecology, University of Alberta, Edmonton, T6G 2S2, Canada; 2 Department of Physiology, University of Alberta, Edmonton, T6G 2H7, Canada; 3 Women and Children's Health Research Institute, Edmonton, T6G 2R3, Canada; University Medical Center Utrecht, NETHERLANDS

## Abstract

Preeclampsia is a disorder affecting 2–8% of all pregnancies, characterized by gestational hypertension (≥ 140/90 mmHg) and proteinuria (≥300 mg over 24 hours) diagnosed following the 20^th^ week of pregnancy, and for which there is currently no available treatment. While the precise cause of preeclampsia is unknown, placental ischemia/hypoxia resulting from abnormal trophoblast invasion and maternal endothelial dysfunction are central characteristics. Preeclampsia is a major cause of both maternal and fetal morbidity and mortality in the perinatal period. In addition, women who have experienced preeclampsia are more likely to suffer cardiovascular disease later in life. The cause of this elevation in cardiovascular risk postpartum, however, is unknown. We hypothesize that there may be lasting vascular dysfunction following exposure to reduced uteroplacental perfusion during pregnancy that may contribute to increased cardiovascular risk postpartum. Using the rat reduced utero-placental perfusion pressure (RUPP) model of preeclampsia, blood pressure was assessed in dams at gestational day 20, one and three months postpartum. Mesenteric artery and aortic function were assessed using wire myography. We demonstrated hypertension and increased mesenteric artery responses to phenylephrine at gestational day 20, with the latter due to a decreased contribution of nitric oxide without any change in methylcholine-induced relaxation. At one month postpartum, we demonstrated a small but significant vasoconstrictive phenotype that was due to an underlying loss of basal nitric oxide contribution. At three months postpartum, endothelium-dependent relaxation of the aorta demonstrated sensitivity to oxLDL and mesenteric arteries demonstrated decreased nitric oxide bioavailability with impaired methylcholine-induced relaxation; indicative of an early development of endothelial dysfunction. In summary, we have demonstrated impaired vascular function following exposure to a RUPP pregnancy that continued into the postpartum period; suggesting that a pregnancy complicated by preeclampsia may predispose women to later life cardiovascular disease via ongoing vascular dysfunction.

## Introduction

Preeclampsia is a disorder of pregnancy characterized by gestational hypertension (systolic blood pressure ≥ 140 mmHg and/or diastolic blood pressure ≥ 90 mmHg) in the presence of proteinuria (≥300 mg over 24 hours) diagnosed following the 20^th^ week of pregnancy [1]. It is a common condition, afflicting an estimated 2–8% of all pregnancies, and is the leading cause of maternal morbidity and mortality [2]. Aside from delivery of the fetus, there is currently no available treatment. While the cause of preeclampsia is unknown, placental ischemia/hypoxia resulting from abnormal trophoblast invasion is a central characteristic, and is associated with the release of many factors including both pro- and anti-angiogenic factors and inflammatory activators that contribute to maternal vascular dysfunction [3–5].

Interestingly, women who have experienced preeclampsia are more likely to suffer cardiovascular disease and cerebrovascular disease later in life [5–7]. Evidence demonstrates an increased risk for hypertension, stroke, ischemic heart disease, and venous thromboembolism [8] and cardiomyopathy [9] following a preeclamptic pregnancy. Notably, women that have had preeclampsia are twice as likely to succumb to cardiovascular-related events than those that have had normal pregnancies [10]. Some investigators have proposed that pregnancy is a vascular stress test, and those women predisposed to cardiovascular disease are revealed through the development of preeclampsia (reviewed in [11, 12]). Alternatively, a preeclamptic pregnancy that develops following placental ischemia may irreversibly modify the maternal vascular system, leaving the mother at increased risk for cardiovascular disease later in life.

Regardless of previous etiology, persistent vascular dysfunction has been observed in women with a history of preeclampsia during the early postpartum period, < three years [13–15], five years postpartum [16] and in those with a history of preeclampsia or pregnancy-induced hypertension at 10–25 years postpartum [17, 18]. In addition, arterial elasticity has been shown to be reduced in women around four years following a preeclamptic pregnancy [19, 20]. Using laser Doppler and iontophoresis, Ramsey *et al*. demonstrated impaired function of both the endothelium and smooth muscle of the skin microvasculature in women at 15–25 years postpartum with a relatively greater effect on the endothelium [18]. Further, circulating levels of noradrenaline have been shown to be increased in women who have had a preeclamptic pregnancy 5–6 years after the event [21]. In the brachial artery, however, endothelial but not smooth muscle function was found to be impaired in the early postpartum period and this could be ameliorated using the antioxidant, ascorbic acid [14].

Animal studies have demonstrated that rats previously exposed to experimentally-induced preeclampsia (infusion of low dose endotoxin) had a heightened blood pressure response to angiotensin II that did not appear to be reflected in aortic vascular reactivity changes at six weeks postpartum [22]. In CD-1 mice, which had been previously exposed to sFlt-1-induced preeclampsia, carotid artery vascular function in response to a range of agonists (acetylcholine, sodium nitroprusside, isoproterenol, phenylephrine, U46619 and 5-hydroxytryptamine) was not found to be altered at 6–8 months postpartum [23]. However, in another study using sFlt-1 treated CD-1 mice, preeclampsia exposure was shown to increase vascular remodeling; including increased smooth muscle cell proliferation and fibrosis [24]. Thus the data in the literature are conflicting and this may be due, in part, to the model used to induce preeclampsia.

The reduced uteroplacental perfusion pressure (RUPP) model of preeclampsia in rats in which placental blood flow is mechanically restricted early in pregnancy has been extensively studied and well-characterized in regard to maternal responses. The RUPP model effectively mimics the systemic effects resulting from placental ischemia in humans including: hypertension, proteinuria, impaired renal function, endothelial dysfunction, increased total peripheral resistance, decreased cardiac output, and fetal growth restriction (reviewed in [25]). Using the RUPP model, we have observed many modifications of the maternal vasculature common to humans, including increased expression of the lectin-like oxidized low density lipoprotein-1 (LOX-1) receptor, increased endothelial NO synthase (eNOS) expression, increased superoxide production, and decreased endothelial function [26, 27]. The LOX-1 receptor is a scavenger receptor that, in our own studies, has been shown to be upregulated in the arteries of women with preeclampsia [28] and can lead to the production of superoxide [29]; suggesting that it may play a role in the development of vascular dysfunction in preeclampsia. We have also identified increased expression of matrixmetalloproteinase-2, in addition to increased vessel contractility in response to big endothelin (bET-1) [30]. Importantly, animal models are not reliant on a maternal predisposition to vascular dysfunction and thus any observed impact on vascular function postpartum is due to the intervention to create a preeclamptic phenotype. Thus, we hypothesize that a preeclamptic pregnancy that develops following placental ischemia can irreversibly modify the maternal vascular system, leaving the mother at an increased risk for cardiovascular disease later in life.

## Methods

### Ethical approval

All protocols were approved by the University of Alberta Health Sciences Animal Policy and Welfare Committee in accordance with the guidelines of the Canadian Council on Animal Care and the Guide for the Care and Use of Laboratory Animals published by the US National Institutes of Health; AUP # 242.

### Reduced uteroplacental perfusion pressure (RUPP) model of preeclampsia

Three-month–old female Sprague-Dawley rats (Charles River, Wilmington, MA) were maintained on *ad libitum* standard rat chow and tap water in a 10:14 hour light:dark cycle. Females were acclimatized in house before breeding. Gestational day 0 (GD0) of pregnancy was determined by the presence of sperm in a vaginal smear following an overnight introduction of a male. On GD14 of pregnancy, rats were anesthetized using inhaled isoflurane (4% induction, 1–3% maintenance) and the abdominal cavity opened by a midline incision. The abdominal aorta was exposed above the iliac bifurcation, and a silver clip (ID 0.230 mm) was placed around the aorta. To prevent compensatory flow via the ovarian arteries, additional silver clips (ID 0.100 mm) were placed around the left and right ovarian arteries between the ovary and the uterine horn. Additional rats underwent a Sham operation, in which comparative manipulations of the arteries were made and silver clips were placed on intra-abdominal fat. Morphine (2 mg/kg) was administered as a postsurgical analgesic to all animals. Surgeries which resulted in hindlimb ischemia (n = 4), indicating severe morbidity, were excluded from the study and the rats were euthanized.

Following surgery, rats were monitored until euthanasia on GD20 (n = 8 Sham and 7 RUPP) or delivery of pups on GD22. RUPP animals that did not have any remaining pups (as a consequence of reabsorption) were excluded from the study and euthanised (n = 15). All litters were culled at birth to control for differences in litter size. Dams were then assessed at either one month (n = 11 Sham and 10 RUPP) or three months (n = 6 Sham and 8 RUPP) postpartum. Evaluation of vascular function in rats at one and three months postpartum was performed to reflect the physiological changes expected within close proximity of pregnancy (early postpartum) and following an extended period of recovery and aging following pregnancy (late postpartum) [31].

### Blood pressure

At their termination date, animals were prepared for in-dwelling blood pressure analysis prior to euthanasia. Rats were anesthetized by inhaled isoflurane and a PTFE #30 catheter inserted into the right carotid artery. Blood pressure was monitored and recorded via an attached pressure transducer (type 379, Hugo Sachs Elektronik, Harvard Apparatus). Heart rate was simultaneously monitored by ECG. The catheter was kept patent via a flush of heparinized saline. After a stable recording of blood pressure and heart rate for ≥10 minutes, animals were euthanized by exsanguination via excision of the superior vena cava.

### Wire myography

Wire myography experiments were performed on arteries from animals at GD20, one and three months postpartum. This study was an extension of previous studies in which we had evaluated certain aspects of aortic and mesenteric function at GD20 [30, 32, 33]. In each set of experiments in the current study, thoracic aortas and/or small 1° or 2° mesenteric arteries were evaluated. Vessels were dissected and cleaned of all surrounding adipose and connective tissues in ice-cold physiological saline solution (PSS), composition (in mM): 10 HEPES, 5.5 glucose, 1.56 CaCl_2_, 4.7 KCl, 142 NaCl, 1.17 MgSO_4_, 1.18 KH_2_PO_4_, pH 7.5). The internal lumen diameters of mesenteric arteries (in μm) were as follows: GD20 Sham 138.0 ± 7.9, RUPP 140.0 ± 5.1; one month postpartum Sham 176.0 ± 4.0, RUPP 184.0 ± 5.0; three months postpartum Sham 170.5 ± 5.1, RUPP 172.8 ± 7.8. The aortic internal lumen diameters (in μm) were as follows: GD20 Sham 1577 ± 43, RUPP 1510 ± 57; one month postpartum Sham 1557 ± 118, RUPP 1708 ± 35; three months postpartum Sham 1537 ± 36, RUPP 1631 ± 24. For each animal, 2 mm segments of mesenteric artery or thoracic aorta were cut and mounted on two 40-μm wires attached to a wire myograph (DMT, Copenhagen, Denmark) to allow isometric tension recordings.

Mesenteric arteries were assessed due to their role as a resistance artery important in the control of systemic blood pressure [34, 35]. Mesenteric arteries were normalized through a series of stepwise increases in diameter to determine their optimal resting tension, set to 0.8 x IC_100_ (the internal circumference equivalent to a transmural pressure of 100 mmHg). Following a 30-minute equilibration period, vascular integrity in all vessels was confirmed by exposing them to phenylephrine (Phe; 10 μM) followed by methylcholine (MCh; 3 μM). Responses to Phe (0.1 to 30 μM) were assessed in the absence or presence of a pan nitric oxide synthase inhibitor (L-NAME; 100 μM). The concentration of Phe producing 80% of the maximum response (EC_80_) was determined for preconstriction prior to assessment of endothelial-dependent relaxation using MCh (0.003–3 μM) in the absence or presence of L-NAME (100 μM) or the prostaglandin H synthase inhibitor indomethacin (5 μM). Lastly, constriction responses to bET were assessed using cumulative concentrations of bET-1 (3–310 nM) in the absence or presence of L-NAME (100 μM). The current manuscript builds considerably on data obtained in previous studies–one of which demonstrated increased responses to bET but not ET at GD20 that was associated with increased matrixmetalloproteinase 2 (MMP-2) expression but not with changes in endothelin converting enzyme (ECE) or endothelin receptor B (ET_B_) [30]. We, therefore, wanted to assess whether the hypersensitivity to bET persisted postpartum as an aspect of the preeclamptic phenotype. Maximum smooth muscle contractility was evaluated at the end of the experiment using a high potassium solution (in mM: 10 HEPES, 5.5 glucose, 4.9 CaCl_2_, 124 KCl, 24 NaCl, 2.4 MgSO_4_, 1.18 KH_2_PO_4_, pH 7.4). Data from mesenteric arteries were presented as force (grams).

Thoracic aortas were assessed as a representative conduit vessel in which increased lectin-like oxidized low density lipoprotein receptor- 1 (LOX-1) expression has been demonstrated in other cardiovascular conditions such as atherosclerosis [36, 37], diabetes [38], hypertension [39] and stress [40] and in the vasculature of women with preeclampsia [29]. Importantly, we have observed increased involvement of the LOX-1 receptor pathway in RUPP dams during late gestation [32, 33]. Thoracic aortas were stretched to 2 grams tension over a series of three adjustments in tension taking a total of 10 minutes. Resting tension was chosen based on published literature [41–44]. Following a 30-minute equilibration period, vascular integrity in all vessels was confirmed by exposing them to Phe (10μM) followed by MCh (3 μM). Responses to Phe (0.1 to 30 μM) were assessed in the absence or presence of L-NAME (100 μM). Endothelial-dependent relaxation was assessed using MCh (0.003–3 μM), following preconstriction of vessels with the EC_80_ concentration of Phe. Responses to MCh were assessed in the absence or presence of L-NAME (100 μM) or oxLDL (10–50 μg/ml). Maximum smooth muscle contractility was evaluated at the end of the experiment using a high potassium solution. Aortic data were presented as force (grams).

All vascular function data were presented as mean ± s.e. Data were summarized as pEC_50_ (negative log of the effective concentration required to produce 50% of the maximum response), E_max_ (maximum response) or area under the curve. Data were analysed using a two-way ANOVA design where the effect of perinatal surgery and inhibitor were determined. A p<0.05 was considered as statistically significant.

### eNOS and LOX-1 receptor expression

Sections of mesenteric artery or thoracic aorta were snap-frozen in liquid nitrogen for subsequent analysis. Western blotting was performed on tissue homogenates using primary antibodies for eNOS (mouse monoclonal antibody, 1:250, BD Biosciences, n = 8–9) and LOX-1 (rabbit polyclonal antibodies, 1:200, Abcam, n = 7–8). Results were normalized to β-actin as a loading control (rabbit polyclonal antibody, 1:1500, Abcam,). Anti-rabbit (IRDye 800 and IRDye 680, 1:20000, Li-Cor) and anti-mouse (Alexa Fluor 750, 1:20000, Invitrogen) secondary antibodies were used. The protein bands were detected and quantified by a Li-Cor Odyssey v3.0 imager system and all data were expressed as percent increase over the corresponding untreated control. All data were presented as mean ± s.e. and analysed by Student’s t-test.

## Results

### RUPP phenotype

Maternal weight at GD0 was not different between any of the Sham or RUPP groups. RUPP surgery led to a similarly reduced litter size in all RUPP groups (Table 1). Correspondingly, maternal weight gain during pregnancy was reduced in the RUPP group (measured at GD20). During the postpartum period, maternal weight was similar between Sham and RUPP animals (measured at one and three months postpartum) and increased with age in both groups (Table 1).

**Table 1:** Maternal and offspring biometrics in experimental groups sacrificed at gestational day 20, one and three months postpartum.

	GD20		1 month postpartum		3 months postpartum	
	Sham	RUPP	Sham	RUPP	Sham	RUPP
	(n = 8)	(n = 7)	(n = 9)	(n = 9)	(n = 6)	(n = 8)
**Maternal weight at GD0 (g)**	285.4 ± 8.1	284.1 ± 6.3	269.9 ± 9.7	284.5 ± 5.8	279.7 ± 18.7	305.6 ± 9.4
**Maternal weight at euthanasia (g)**	400.0 ± 20.5	316.8 ± 13.1***	326.8 ± 6.7	329.8 ± 6.8	415.1 ± 1.1	387.4 ± 23.0
**Viable pups**	16.1 ± 0.6	4.6 ± 1.1***	11.7 ± 0.5	5.8 ± 1.9**	11.8 ± 0.8	7.3 ± 1.6*
**Pup weight (g)**^**a**^	3.6 ± 0.1	3.2 ± 0.1*	7.0 ± 0.1	6.1 ± 0.2***	7.0 ± 0.2	6.0 ± 0.2**
**Crown-rump length (mm)**^**a**^	42.4 ± 0.5	39.9 ± 0.6**	43.5 ± 0.8	41.0 ± 1.2	43.3 ± 0.5	46.9 ± 1.5
**Abdominal girth (mm)**^**a**^	39.9 ± 0.3	37.7 ± 0.6**	44.6 ± 0.9	41.6 ± 0.9*	44.7 ± 0.4	43.6 ± 1.1
**CR/AG ratio**	1.06 ± 0.01	1.06 ± 0.01	0.98 ± 0.02	0.98 ± 0.02	0.97 ± 0.01	1.05 ± 0.05

^a^ Pup biometrics were taken at either GD20 or GD22 (one and three month postpartum groups). GD: gestational day, CR: crown-rump length, AG: abdominal girth. Data were analysed by t-test within each maternal group (GD20/one month postpartum/three months postpartum).

*: p<0.05

**: p<0.01

***: p<0.001.

**Fig 1:**
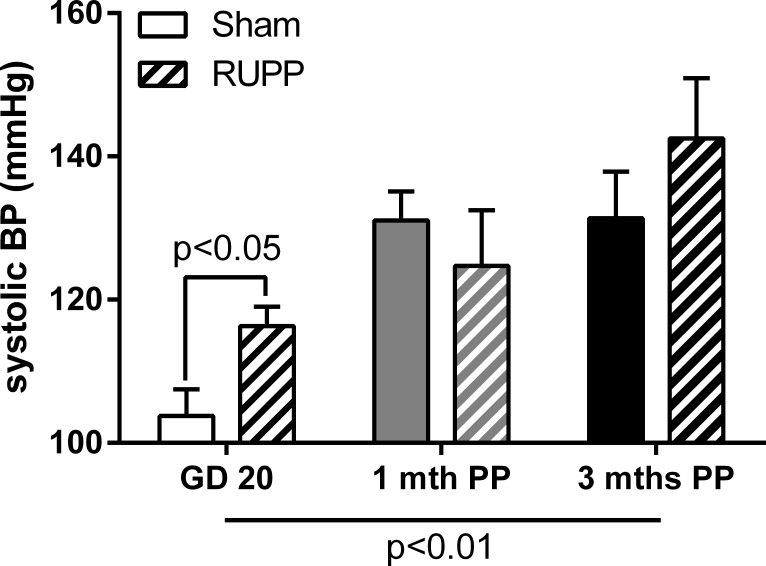
Systolic blood pressure in Sham and RUPP groups pre- and postpartum. Systolic blood pressure was increased in the RUPP group compared to Sham at GD20 but not at one or three months postpartum. Data were analysed by two-way ANOVA with group (perinatal surgery or time point) effects and post-hoc t-test, n = 3–9/group.

Systolic blood pressure was increased in the RUPP group at GD20 (Fig 1, p = 0.01) but not at one month postpartum (p = 0.53) or three months postpartum (p = 0.40). While blood pressure appeared to continue to rise at three months postpartum (142.5 ± 8.4 mmHg vs. 124.7 ± 7.7 mmHg at one month postpartum), this did not reach significance. Neither diastolic blood pressure nor heart rate was different between the groups at GD20 or either postpartum stage.

Offspring of all RUPP pregnancies were growth restricted; weight measurements taken at GD20 and at birth were lower compared to Sham litters (Table 1). In addition, RUPP surgery had effects on reducing abdominal girth (GD20 and one month postpartum groups) and crown-rump length (GD20 group), but not the crown-rump length/abdominal girth ratio, suggesting symmetrical growth restriction in RUPP offspring.

### Mesenteric artery vasoconstrictor function

**Fig 2:**
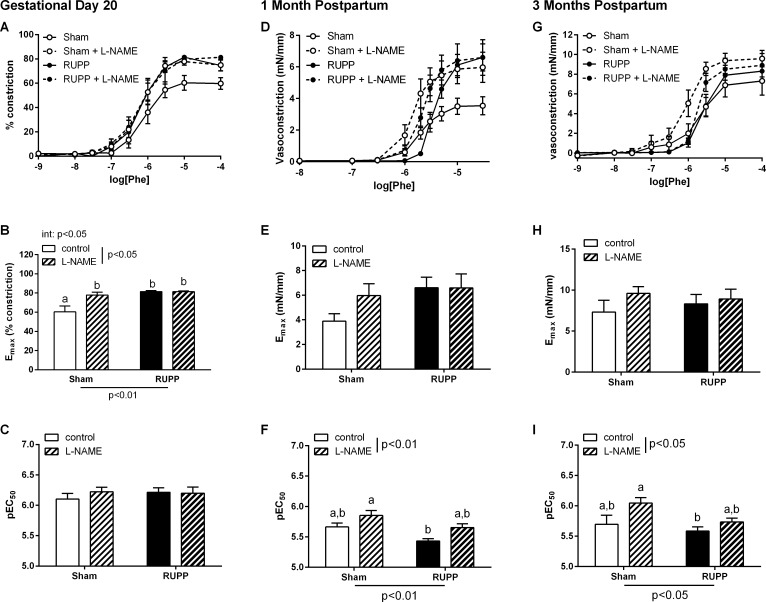
Mesenteric artery constrictor responses to phenylephrine (Phe). Maximal constrictor responses to Phe were increased in vessels from RUPP compared to Sham at gestational day (GD) 20. At one and three months postpartum there was a decreased sensitivity to Phe in the RUPP group. **A, D and G**: vasoconstrictor concentration-response curves in Sham or RUPP in the absence or presence of the nitric oxide synthase inhibitor, L-NAME (100 μM). **B, E and H**: summary data of maximal constrictor responses in each group. **C, F and I**: summary pEC_50_ data in each group. Data were analysed by two-way ANOVA with group (perinatal surgery or inhibitor) or interaction (int) effects, different letters denote significant differences by Bonferroni post-hoc testing, n = 5–9/group.

In the mesenteric artery, maximal vasoconstrictor responses to Phe were increased at GD20 (Fig 2A–2C, E_max_ p<0.01) in RUPP vs Sham animals and this was due to a reduction in basal NO since responses in Sham, but not RUPP, vessels were increased in the presence of L-NAME (Fig 2A–2C, effect of L-NAME: p<0.05, interaction: p<0.05). While this pattern appeared to continue until one month postpartum (Fig 2D–2F), the data were more variable and, therefore, the effect did not reach significance in regard to effects on maximal vasoconstriction. Interestingly, however, the sensitivity of the mesenteric arteries to Phe was increased by the presence of L-NAME (Fig 2F, effect of L-NAME p<0.01), where it had been unchanged at GD20 (Fig 2C). Notably, there continued to be a group effect such that NO involvement was reduced in RUPP compared to Sham (Fig 2F, effect of group p<0.01). By three months postpartum, responses to Phe were normalized in RUPP vessels with no significant difference in the maximum responses to Phe in Sham and RUPP vessels (Fig 2G–2I). A similar pattern of altered sensitivity in the presence of L-NAME (as observed at one month postpartum) emerged (Fig 2I) whereby there was an inhibitor effect (p<0.05) that was reduced in RUPP vs. Sham vessels (p<0.05).

**Fig 3:**
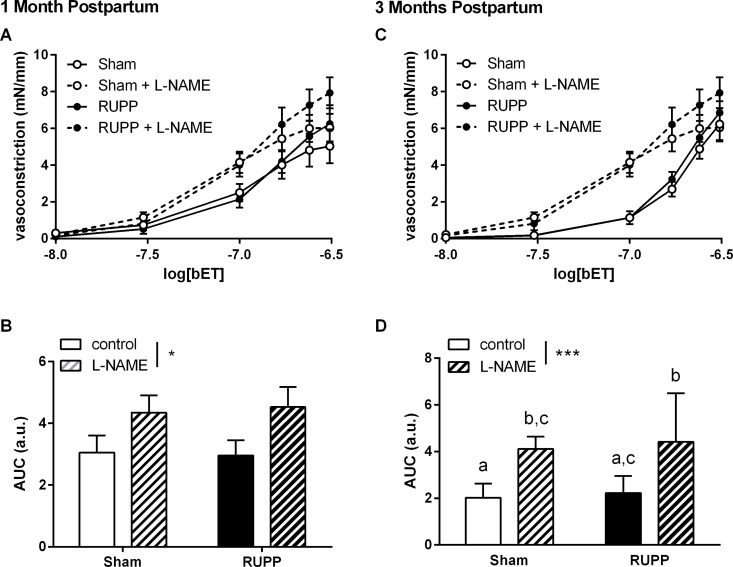
Mesenteric artery constrictor responses to big endothelin (bET). Constrictor responses to bET were unaltered in vessels from Sham and RUPP animals at one and three months postpartum. **A and C**: vasoconstrictor concentration-response curves in Sham or RUPP in the absence or presence of the nitric oxide synthase inhibitor, L-NAME (100 μM). **B and D**: summary area under curve data in each group. Data were analysed by two-way ANOVA with group (perinatal surgery or inhibitor) or interaction (int) effects, different letters denote significant differences by Bonferroni post-hoc testing, n = 6–9/group.

We have previously published results demonstrating that mesenteric artery responses to bET, but not ET-1, were increased at GD20 in vessels from the RUPP group [30]. In the current study, vasoconstrictor responses to bET were unaltered at one (Fig 3A and 3B) and three (Fig 3D and 3E) months postpartum. In the presence of L-NAME, responses to bET were increased in both Sham and RUPP groups (Fig 3B, effect of L-NAME p<0.05) and this effect appeared to increase with age (Fig 3D).

Constrictor function assessed by maximal constriction to KPSS was unaltered at one (E_max_: Sham 4.73 ± 0.69 mN/mm vs. RUPP 5.76 ± 0.59 mN/mm) and three (E_max_: Sham 1.51 ± 0.22 g vs. RUPP 1.57 ± 0.17 g) months postpartum in the mesenteric artery; as has been previously observed at GD20 [30].

### Mesenteric artery relaxation function

**Fig 4:**
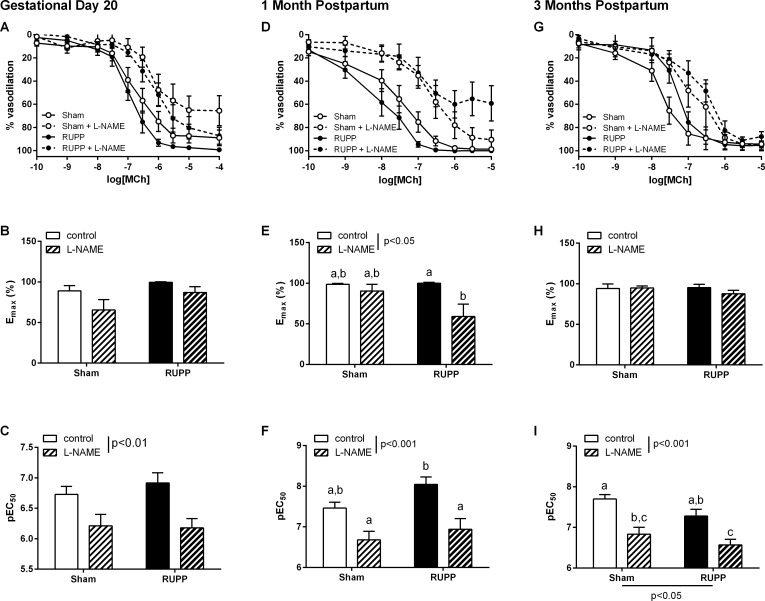
Mesenteric artery dilator responses to methycholine (MCh). Dilator responses to MCh were unaltered in vessels from Sham and RUPP animals at gestational day (GD) 20 and one month postpartum. At three months postpartum, there was a decreased sensitivity to MCh in the RUPP group. **A, D and G**: vasodilator concentration-response curves in Sham or RUPP in the absence or presence of the nitric oxide synthase inhibitor, L-NAME (100 μM). **B, E and H**: summary data of maximal constrictor responses in each group. **C, F and I**: summary pEC_50_ data in each group. Data were analysed by two-way ANOVA with group (perinatal surgery or inhibitor) or interaction (int) effects, different letters denote significant differences by Bonferroni post-hoc testing, n = 4–8/group.

Relaxation of the mesenteric artery to MCh was not altered by RUPP surgery at any stage: GD20, one or three months postpartum (Fig 4A–4I). Indomethacin did not affect relaxation responses to MCh in either Sham or RUPP groups at any age (p<0.05). L-NAME inhibited MCh-induced relaxation at GD20 (Fig 4C, effect of L-NAME p<0.01), one month postpartum (Fig 4F, effect of L-NAME p<0.001) and three months postpartum (Fig 4I, effect of L-NAME p<0.001) in both Sham and RUPP vessels. While there was no significant difference in the effect of L-NAME between the Sham and RUPP groups at GD20 or one month postpartum, at three months postpartum relaxation to MCh was reduced in the RUPP group (Fig 4I, group effect: p<0.05).

### Mesenteric artery eNOS expression

**Fig 5:**
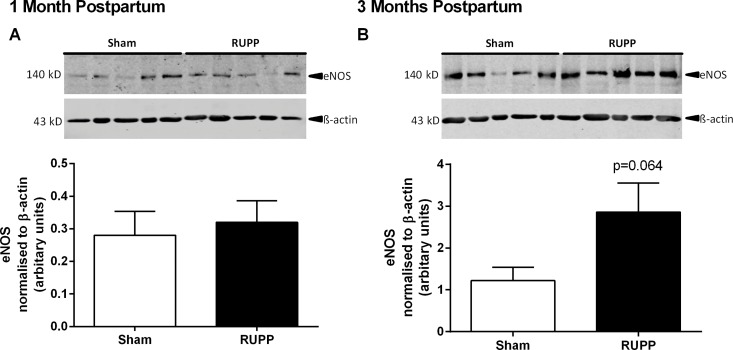
Endothelial nitric oxide synthase (eNOS) expression in mesenteric arteries. Expression of eNOS in vessels from Sham and RUPP animals was unaltered at one month postpartum (A) but tended to be increased at three months postpartum (B). All data were normalised to β-actin. Representative blots from a single gel are shown, n = 5/group.

To further investigate the involvement of NO in vascular function, eNOS expression was determined in the mesenteric arteries. We have previously shown that eNOS expression was increased at GD20 in mesenteric arteries [30] (and aorta [32]). In the current study we observed unaltered eNOS expression at one month postpartum while there was a tendency (p = 0.064) towards increased eNOS expression at three months postpartum (Fig 5). Neither phosphorylated eNOS (Ser 1177) nor eNOS dimers were present at sufficient levels for analysis (data not shown).

### Thoracic aorta vasoconstrictor function

Similar to our previously published data at GD20 [27, 32], aortic vasoconstrictor responses to Phe were not altered by prior RUPP surgery at one month (E_max_: Sham 0.93 ± 0.11 g vs. RUPP 0.96 ± 0.14 g) or three months (E_max_: Sham 1.05± 0.16 g vs. RUPP 1.35 ± 0.12 g) postpartum. Further, constrictor responses to Phe in both groups were increased similarly in the presence of L-NAME at both postpartum stages (group effect of L-NAME: p<0.01). Constrictor function, as assessed by maximal constriction to KPSS, was also unaltered at one (E_max_: Sham 1.11 ± 0.22 g vs. RUPP 1.11 ± 0.13 g) and three (E_max_: Sham 1.64 ± 0.34 g vs. RUPP 1.67 ± 0.17 g) months postpartum in the thoracic aorta.

### Thoracic aorta relaxation function

**Fig 6:**
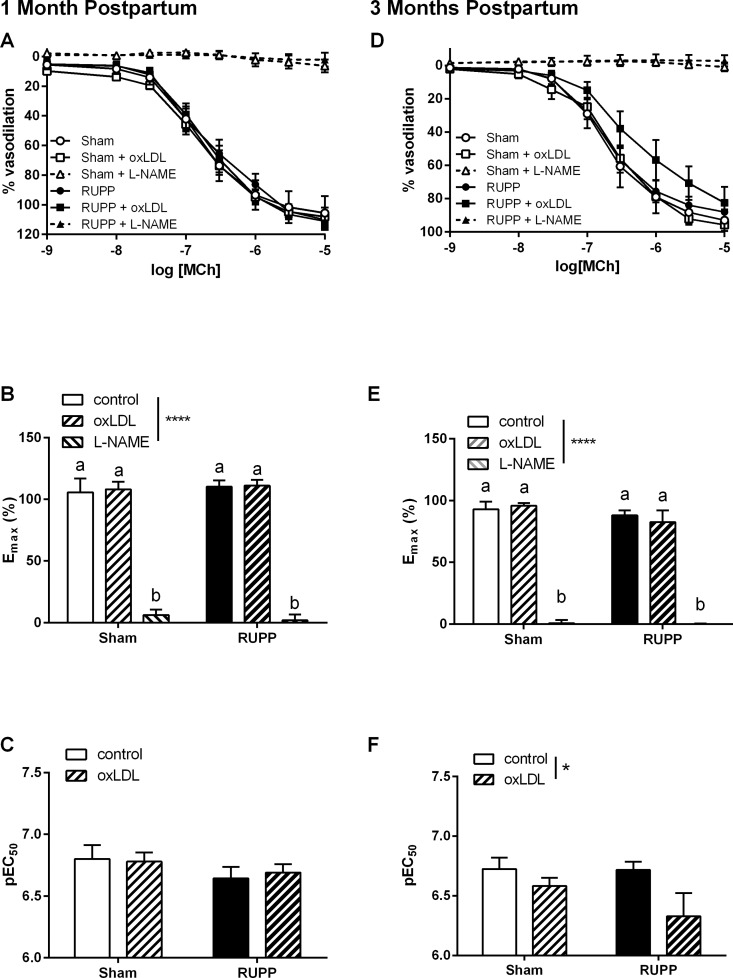
Thoracic aorta dilator responses to methycholine (MCh). Dilator responses to MCh in vessels from Sham and RUPP animals were unaltered at one month postpartum but demonstrated a decreased sensitivity to MCh in the presence of oxLDL at three months postpartum. **A and D**: vasodilator concentration-response curves in Sham or RUPP in the absence or presence of the nitric oxide synthase inhibitor, L-NAME (100 μM) or oxidized LDL (10–50 μg/ml). **B and E**: summary data of maximal dilator responses in each group. **C and F**: summary pEC_50_ data in each group. Data were analysed by two-way ANOVA with group (perinatal surgery or inhibitor) or interaction (int) effects, different letters denote significant differences by Bonferroni post-hoc testing, n = 5–8/group.

In our previous studies on the RUPP model of preeclampsia, the effect on aortic MCh-induced relaxation has been slight with either no change [33] or a subtle decrease [32] in the relaxation response at GD20. In both cases, however, we discovered an increased involvement of oxLDL in the RUPP group via increased expression of the LOX-1 receptor [32] and a direct impact on vasodilator function [33]. In the current study we observed that MCh-induced vasodilator function was unaltered by prior RUPP surgery at either one (Fig 6A–6C) or three (Fig 6D–6F) months postpartum. At all ages, aortic vasodilator responses to MCh were shown to be primarily due to NO since relaxation was completely abolished by L-NAME (Fig 6, effect of inhibitor p<0.0001). Interestingly, at three months postpartum, but not one month postpartum, the inclusion of oxLDL reduced relaxation; particularly in the RUPP group (Fig 6F, group effect of oxLDL p<0.05).

### Thoracic aorta LOX-1 receptor expression

**Fig 7:**
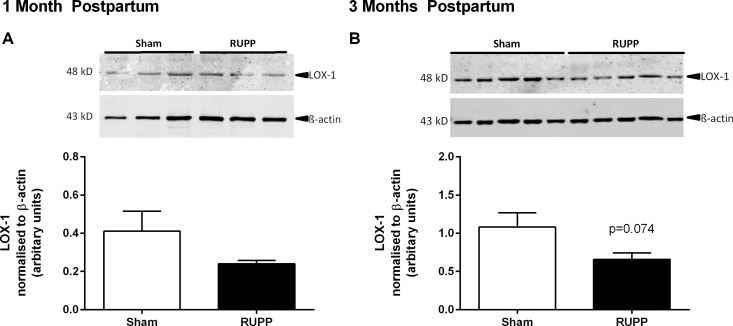
Lectin-like oxidised LDL (LOX-1) receptor expression in thoracic aorta. Expression of LOX-1 in vessels from Sham and RUPP animals were unaltered at one month postpartum (A) and tended to be decreased at three months postpartum (B). All data were normalised to β-actin. Representative blots from a single gel are shown, n = 3–5/group.

To further investigate the involvement of the LOX-1 receptor in vascular function we determined the aortic LOX-1 expression levels. In line with our vascular function studies at GD20, we observed increased aortic LOX-1 expression at this time point [32]; however, this was not confirmed in a follow up study [33]. When we investigated LOX-1 expression at one month postpartum, we did not observe any difference between the Sham and RUPP groups (Fig 7A). However, at three months postpartum, LOX-1 expression tended to be decreased in RUPP compared to Sham aorta (Fig 7B, p = 0.074).

All raw data is provided in S1 File.

## Discussion

In the current study we used an animal model of preeclampsia to address persistent alterations in vascular health during late gestation and the postpartum period; building on data which we have previously published using the RUPP model of preeclampsia [30, 32, 33]. We demonstrated vascular dysfunction and hypertension at GD20 that was largely resolved in the early postpartum period. At a later postpartum stage some indices of vascular dysfunction were present that, combined with an indication that blood pressure might also be continuing to rise, suggest a predisposition to cardiovascular dysfunction following exposure to reduced placental perfusion during gestation. This model has been well established (reviewed in [25, 45]) and is one of the few models of preeclampsia which relies on reduced perfusion to the utero-placental units instead of targeting a specific pathway (for example sFlt or L-NAME) and which does not have preexisting hypertension. As such, the phenotype of the RUPP model develops secondary to an underperfused placenta; as occurs in women with preeclampsia (reviewed in [46]).

Consistent with our previous work [32, 33], in the current study systolic blood pressure was elevated at GD20 in RUPP animals. Interestingly, the elevated blood pressure in the RUPP group appeared to be the result of a lack of the normal hypotensive response to pregnancy [47] which was observed in the Sham group. In both groups blood pressure increased in the postpartum period; resolving the difference between the groups at both one and three months postpartum. Since delivery of the placenta is also known to resolve preeclampsia in women (reviewed in [46]), this further confirms the model of placental dysfunction in this model. Blood pressure in the RUPP group appeared to continue to increase in the postpartum period while the Sham group remained stable. The observed effects on maternal weight, litter size and pup growth suggest that the severity of the RUPP phenotype was equivalent between the GD20, one month and three month postpartum RUPP groups; excluding this as a factor in the slightly increased postpartum blood pressure at three months.

In the current study we further defined the vascular phenotype during late gestation. While we had previously shown in the thoracic aorta unaltered constrictor (response to PE) and subtly decreased dilator (MCh-induced) responses that were associated with underlying alterations in the oxLDL/LOX-1 receptor pathway, the mesenteric arteries expressed a very different phenotype. In addition to previously observed increases in constriction to bET, in this study we demonstrated increased responses to Phe that were due to a decreased contribution of NO. While this would lead to the expectation that dilator responses might also be reduced, we did not observe any difference in the MCh-induced endothelium-dependent relaxation in this group. Thus, despite there being a maintained vasodilator capacity, there was a distinct mesenteric artery constrictor phenotype. Since the mesenteric arcade can play a significant role in the control of blood pressure, given its ability to accommodate approximately 25% of the blood volume [34, 35], an imbalance in favor of constriction in this vascular bed might contribute to the hypertension observed at this time point.

Hypertension has been demonstrated to resolve in the postpartum period in women with preeclampsia [48], likely due to the absence of the placenta and circulating factors. However, exposure to a preeclamptic pregnancy has been shown to increase the risk of later development of cardiovascular disease [5–7]; suggesting an ongoing effect on postpartum health. In the current study we aimed to determine the potential vascular pathways by which the effects of placental ischemia could persist postpartum. During the early postpartum stage we demonstrated that aortic vasodilator and vasoconstrictor function was unaltered in RUPP compared to Sham animals. In mesenteric arteries, however, there was a subtle vasoconstrictive phenotype that was seen as increased reactivity to adrenergic agonists and was due to an underlying loss of basal NO contribution; potentially a persisting phenotype from the late gestational stages. At this stage, therefore, the vascular function appeared to have almost completely returned to normal. In accordance with the vascular function parameters, both mesenteric eNOS expression and aortic LOX-1 expression had returned to control levels by one month postpartum.

At a later postpartum stage, however, endothelium-dependent relaxation of the aorta demonstrated sensitivity to oxLDL that was not apparent at one month postpartum but was reflective of the gestational phenotype. Further, in the mesenteric arteries, while total constrictor and dilator capacities were unaltered there was indication of decreased basal NO contribution to both of these functions. It is possible that these indications of an increased involvement of the LOX-1 receptor and decreased NO bioavailability might be the early development of endothelial dysfunction. While LOX-1 receptor expression tended to be decreased in the postpartum period, this may reflect increased activation and subsequent internalization/recycling of the receptor. It is interesting to note that these changes occurred in animals which appeared to be otherwise fully recovered at an earlier time point (one month postpartum) but had been exposed to no further adverse events beyond normal aging. We speculate that additional co-morbidities associated with aging in humans, such as obesity, would exacerbate vascular dysfunction that was initiated during a preeclamptic pregnancy.

The function of both aortic and mesenteric preparations were investigated due to their relative importance in the development expression of LOX-1 associated with cardiovascular disease (aorta) and in blood pressure control (mesenteric). The LOX-1 receptor is a scavenger receptor that has been shown to be expressed in vessels in both women with preeclampsia and in animal models of the disorder [26, 29, 49]. Further, the production of oxidized products such as oxidized lipoproteins (oxLDL) has also been shown to be increased in preeclampsia [50, 51]. In our studies we have also shown that the LOX-1 receptor plays a role in vascular dysfunction both during a RUPP gestation and in the late postpartum period; suggesting that it may be an indicator of developing vascular disease and may be central to the increased cardiovascular risk in the postpartum period. Interestingly, although the aorta is a conduit artery that has little influence on blood pressure, during the RUPP gestation the aorta displayed a constrictive phenotype that was also seen in the mesenteric arteries; suggesting a systemic response to factors which might mediate the hypertensive effects of a low placental perfusion. Vascular dysfunction was further evidenced in the mesenteric arteries at GD20 and this became largely resolved at one month postpartum, along with a resolution of blood pressure to control levels. Aspects of dysfunction persisted at three months in both the mesenteric and aortic vessels suggesting that the vasculature did not fully recover from the insult of preeclampsia and may remain at increased risk for cardiovascular disease in later life. It is possible that the acute effects of an ischemic placenta during pregnancy caused an underlying, subclinical pathology that only began to develop into a cardiovascular dysfunction phenotype with the passing of time. Continuing the model to a later postpartum stage might reveal a continued increase in blood pressure with age. In the current study we defined some of the vascular function pathways involved in postpartum cardiovascular dysfunction; however, additional studies will be necessary to assess whether other aspects, such as endothelial damage, vascular stiffness, and oxidative stress, are involved in the ongoing cardiovascular risk following preeclampsia. Using these approaches, potential therapeutic options may be identified and developed.

In summary, the prior exposure of pregnant animals to a preeclamptic-like condition altered their vascular physiology in such a way that it was less able to maintain normal function at three months postpartum. Importantly, in using an animal model, the genetic background and environmental exposures of the normal pregnant and RUPP animals were identical; thus we are able to attribute our findings solely to the differences in their pregnancies. We have demonstrated impaired vascular function following exposure to a RUPP pregnancy that continues into the postpartum period, suggesting that a pregnancy complicated by preeclampsia may predispose women to later life cardiovascular disease.

## Supporting Information

S1 File Raw Data. (ZIP)
